# On Carcinoma in Cattle1Read before the Chicago Academy of Medicine, Feb. 9, 1900.

**Published:** 1900-07

**Authors:** Leo Loeb, George Jobson

**Affiliations:** Assistant Professor of Pathology in the Chicago Policlinic; Government Meat-Inspector


### THE JOURNAL
OF
### COMPARATIVE MEDICINE AND VETERINARY ARCHIVES.

VOL. XXI. *JULY, 1900.* No. 7.

### ON CARCINOMA IN CATTLE.[1]

BY LEO LOEB, M.D.,
ASSISTANT PROFESSOR OF PATHOLOGY IN THE CHICAGO POLICLINIC,
AND
GEORGE JOBSON, D.V.S.,
GOVERNMENT MEAT-INSPECTOR.

PREPARATORY to certain investigations on carcinoma, we first had to examine the occurrence of carcinoma in cattle. Inasmuch as it seems to us that they may be of some general interest, we will give a short account of a few of the results obtained.

In looking over the special works on the pathology and therapeutics of domestic animals, we find but little on carcinoma. In Kitt's *Pathological-anatomical Diagnosis* (Stuttgart, 1894) we have thus far found the best record of the reported cases of carcinoma. We find relatively a small number of isolated cases of carcinoma of cattle reported, usually only a single case in each instance, of carcinoma of the mammary gland, of the gall-bladder, perhaps of the second stomach, and carcinomatous ulcer of the first stomach of cattle, carcinoma of the bladder, ovary, and uterus, and also adenocarcinoma of the kidney. Among these cases only a certain number seem to have been carefully examined microscopically. In a special work by G. Woodruffe Hill (London, 1881), entitled *Bovine Medicine and Surgery*, we find, under the designation "Cancer," various tumors, like carcinoma and sarcoma, undifferentiated. Thus he describes a cancer of the maxilla, which, according to illustration and description, might perhaps as well be a periosteal sarcoma as a carcinoma. His description of cancer of the eye, called fungus hæmatodes, so far as

[1] Read before the Chicago Academy of Medicine, Feb. 9, 1900.

its macroscopic appearance is concerned, is incorrect. The tumor is said to originate behind the eye, causing it to protrude, but we will presently see that the origin and development of the tumor is of quite a different type. There is no mention made of microscopic examination.

Our results are based on the statistics of the cases of carcinoma in cattle at the Stock Yards since July, 1898, in which the principal organs were examined postmortem. In sixteen cases, from September, 1899, to February, 1900, a fuller postmortem examination was made. In a number of instances in the cases last mentioned, microscopical examinations were made of such organs and lymphatic glands in different parts of the body as had a suspicious appearance. Our present report sets forth only the results thus far obtained. We hope to collect additional material.

The first question is: Where do we find carcinoma in cattle? So far we have found it in two places, viz., at the inner canthus of the eye and on the vulva. We must add, however, that all the cases thus far seen, with the exception of one carcinoma of the vulva, were carcinomata of the inner canthus of the eye. Therefore, by far the largest majority of cases of carcinoma we find in this one place. Just as we have found one case of carcinoma of the vulva, we may expect to find an occasional carcinoma at some of the other places mentioned in literature. But it is important to emphasize the predominance of carcinoma at this one point, as shown by our examinations, as far as they have gone up to the present. Further investigations may possibly modify our results to some degree.

The second question is: How often do we find carcinoma in cattle? The following tabulated statement will give us information on this point:

From July, 1898, to January, 1899, but four cases were seen. These were observed during the month of October. No case in November and December, 1898. From January 1, 1899, to January 1, 1900, we counted forty-eight cases of carcinoma.

The following is a monthly statement of the number of cases examined: January, 1899, none; February, 6; March, 3; April, 2; May, 2; June, none; July 3; August, 3; September, 4; October, 2; November, 9; December, 14; total, 48.

So we find in one year (1899) forty-eight cases at the Stock Yards. In addition there have been in the last two years two other cases of carcinoma reported at the Stock Yards, but which were inaccessible to examination. That would bring the number up to forty-nine cases in one year. In the same year 2,514,446 head of cattle were received at the Stock Yards, so that there was one carcinomatous animal in every fifty thousand. Of course, this can only give an approximate idea of the frequency of carcinoma, and only upon the supposition that as many carcinomatous cattle are delivered at the Stock Yards as non-carcinomatous cattle. This may easily be the case, the owner of the carcinomatous cattle getting some pay also for the diseased cattle, and in this way running no risk of losing all, should the animal die.

Concerning the sex of diseased animals, there is one fact which at first glance seems very remarkable. Of the sixty animals examined, fifty-nine were cows, and only one animal was a steer, about six years old. This one steer was perfectly white. It is indeed a fact that animals without pigment behave in certain ways differently from pigmented animals; thus we find melanotic sarcomata, as is well known, usually in white horses. But this apparently remarkable fact touching the distribution of sexes will be explained, if we take into account the circumstances that steers are usually killed when young, often before the sixth year—the majority of them when quite young—the cows becoming much older. All the cows coming to the Stock Yards with carcinoma were from six to fifteen years of age, therefore of greater age than steers usually attain. Still we cannot absolutely deny the possibility that sex may have something to do, after all, with the frequency of carcinoma in cattle. It is, however, much more probable that the remarkable findings in this respect are to be explained by the difference in age. Thus it seems that carcinoma in cattle, as in man, is a disease of advanced years of life.

How does the development of carcinoma affect the general state of health of the animal? All the animals affected with carcinoma were very much emaciated. This loss of weight was already present when the carcinoma was not very much ulcerated. The emaciation was most pronounced in the animals most affected by carcinoma. Metastases in the inner organs had nothing to do with this effect, as we shall see later on. But how far this effect is to be attributed to carcinoma, and how far to ulceration (occurring also in the beginning, but in a slight degree), it is difficult to state.

We made a number of inquiries as to the rapidity of development of carcinoma, the external circumstances that might influence the disease, and the first changes visible. Most of our attempts to get information from the proprietors have been unsuccessful thus far, but through the kindness of Dr. Holcombe our attention was directed to a cattle ranch near Cheyenne, Wyoming, whence we received some information. Here is a ranch which keeps about a thousand cattle, about two thousand coming and going during the year. On this ranch were found every year one or two cases of carcinoma of the eye. This observation covers a period of ten years, as the proprietor informed us in answer to our written inquiries. According to Dr. Holcombe, who lived in this neighborhood as military veterinarian, the ranches in the adjoining district of equal size are practically free from this disease. Thus it seems that carcinoma of the inner canthus of the eye is almost endemic on this one ranch. That the disease is not very rapid can be inferred from the statement of the proprietor of this Wyoming ranch, that pregnant carcinomatous cows are expected to give birth to calves. The first symptom noticed is what the proprietors call a "running eye."

What is, then, the development of this carcinoma of the eye, known among veterinarians under the name of fungus hæmatodes? We saw two cases where the lesion was still in a comparatively early stage. We will describe such a case. In the region of the caruncula, which in normal animals is about the size of a pea, rather deep-seated, just between the opening of the lachrymal ducts, and covered with hairs, we see a papillary elevation about the thickness of a finger. The membrana nictitans, which is very large in cattle, and is pushed forward laterally when a foreign body is in the eye, was slightly thickened, very little ulcerated, the ulceration being barely visible to the naked eye. The conjunctiva of the lid was almost normal, but near the inner canthus could be seen a number of small nodules, which pushed forward the conjunctiva in a few places. There was a slight ulceration of the mucous membrane of the conjunctiva. The skin around it was normal, as always in young cases.

A case further advanced presents the following picture: In the region of the inner canthus we see large tumor masses, which appear papillomatous, especially on sectional view. They are ulcerated on the surface, and covered with the remnants of old hemorrhages. The lower and upper lids, especially on the conjunctival side, are ulcerated, and sometimes partially grown together. The eye is displaced latterally and backward. The cornea is opaque. If one opens the orbital cavity, one will find tumor masses in the orbital fat. If the carcinoma is already far advanced, it penetrates into the bone, and may break through in different directions. For instance, we may see the tumor penetrate into the antrum of Highmore, as a round body covered with small protruding nodules that indicate the direction of growth. In some cases we may also observe how the carcinoma begins to penetrate into the eye. We observe this at the cornea, as well as at the junction of the cornea with the sclera, and likewise further backward into the sclera toward the choroidea. Later on the eye will be entirely destroyed. Out of a total of thirty-two cases we found the eye destroyed in six, and the bone affected in fifteen.

What do we find with reference to metastases? In thirty-two cases, where the lymphatic glands and the inner organs were more carefully examined with this point in view, we found the retromaxillary gland affected twenty times, and as a rule changed into a large metastatic tumor with some remains of lymphatic tissue; twelve times, viz., in the twelve least advanced cases, we found the lymphatic glands free of metastases. The macroscopic findings were confirmed in a number of cases by microscopic examination. Once we found the submaxillary gland, once the retropharyngeal gland, once the anterior mediastinal gland, similarly enlarged and changed. In these last three cases it was impossible to examine the glands microscopically. Once we found a metastatic infiltration in the masseter muscle of the same side. We also examined microscopically a number of lymphatic glands in other parts of the body—cervical, submaxillary, bronchial, mediastinal, portal, and mesenteric glands, with somewhat suspicious appearance—always, however, with negative results. Similarly we could find neither macroscopically nor microscopically any metastasis in the inner organs. Although we found extremely large metastases formed in the retromaxillary gland, even in the majority of cases, we found no further metastasis, with the exception of the few cases mentioned, where probably a neighboring gland was affected. In one case where we were induced to microscopically investigate the lungs with the bronchial glands, we found in both signs of tuberculosis—

necrotic tissue and typical giant-cell tubercles. Tubercle bacilli were not demonstrated, as bacterial stains were not employed; but the structure of the giant cell and its connection with the surrounding tissue exclude a foreign body as the exciting cause. To this combination of tuberculosis and carcinoma in cattle we shall also give attention in further investigations.

Two cases of carcinoma among the sixty investigated excited our special interest, because in these cases there were present in the same animal two carcinomata, the carcinoma of the vulva, already mentioned, and in the second case the inner canthus of both eyes was affected with carcinoma, the disease being further advanced in one eye than in the other. In the case of carcinoma of the vulva (which, by the way, was more ulcerated than the carcinoma of the eye) we found by macroscopic and microscopic examination distinct carcinomatous nodules beneath the mucous membrane. But the inguinal lymph glands in this case were microscopically found free from carcinoma. If we consider how rarely, relatively speaking, carcinoma occurs in cattle (according to our investigations about one case in fifty thousand), the occurrence of a double carcinoma in two of the sixty cases is surprising. Whether we are to regard this as an autoinoculation in the case of the vulvar carcinoma, possibly caused by the rubbing of the head at the vulva, or whether there were certain constitutional conditions necessary for the occurrence of carcinoma which made multiple occurrence easy, must at present be left undecided.

Histological examination showed the carcinoma of the eye to be one of stratified epithelium with horny changes and epithelial pearls, as they can be observed in carcinoma of stratified epithelium from various other places. The metamorphosed epithelium showed less horny fibres, or lamellæ, than compact horny layers, such as take up eosin very readily. This character was most pronounced in the cases not very far advanced, in which we found a papillomatous elevation in the region of the caruncula. Here we found stratified epithelium arranged around the hairs and then penetrating into the deeper tissues, forming horny masses in the centre of the alveoli, red-stained with eosin. In other cases already further advanced we were never able to see anything of the caruncula.

If we follow microscopically the development of the carcinoma, we find in comparatively fresh cases the carcinoma in the deeper part of the membrana nictitans. In many cases we are able to see the mucous membrane of the membrana nictitans intact over the tumor. In other places probably a secondary union of the tumor and mucous membrane has taken place. In still other places the mucous membrane is thrown off, and the growing carcinoma takes its place. Often in these cases it is difficult to see where the tumor begins and the mucous membrane ends.

In the connective tissue of the membrana nictitans at the side of the hyaline cartilage, which lies in the centre of this fold, we find a rather large gland, the so-called Harder's gland, which penetrates deep into the orbital cavity. In no case was it possible to see the transition of this gland into the tumor. Both were separated by connective tissue, or possibly the tumor had entirely replaced one lobule of the gland. The glands sometimes showed degenerative changes. We find occasionally the remains of a gland duct in the tumor tissues under these conditions. Other small glands in this region are also occasionally surrounded by the tumor, but there is no indication that the tumor originates from these glands. That supposition would be excluded, furthermore, by the histology of the tumor, as above described.

In the orbital cavity we also find the glandular nodules and the nodules of the tumor sharply separated. From thence the tumor penetrates further beneath the conjunctiva of the upper and also of the lower lid. The relation of the tumor to the mucous membrane of the conjunctiva at this place is similar to that in the membrana nictitans. Often one can see distinctly beneath the mucous membrane a layer of lymphocytes, and beneath these the tumor, but in other places the pressure of the tumor destroys the mucous membrane. The carcinoma may then grow up and include the remains of the mucous membrane.

If the tumor penetrates into the eye we find at some places the chromatophores of the iris and choroidea between the alveoli of the tumor. Frequently the tumor is surrounded by small round cells, which show at the side of the tumor not infrequently the character of plasma cells—just as the mucous membrane of the conjunctiva, which lies near the tumor, is accompanied by lymphocytes.

The bloodvessels near and between the tumor masses are often affected by endarteritis. The connective tissue, especially in metastases of the lymph glands, we find occasionally tendon-like. The tumor itself, during its progressive penetration into the surrounding tissues, retains on the whole its character; but sometimes we find, instead of the horny changes inside of the alveoli, loosely arranged hyaline, carcinomatous cells. The character of the tumor also remains unchanged in the metastases of the lymph glands. At some places we see the tumor cells penetrating between the lymph cells.

These histological findings with macroscopical observations make it at least very probable that the tumor originates from the epithelium of the caruncula or the conjunctival epithelium just surrounding the caruncula. In our future investigations we shall endeavor to find, if possible, still earlier stages, and if these should modify our conclusions we shall communicate it.

The carcinoma of the vulva shows on the whole the same character as the carcinoma of the eye. But this alone does not warrant the conclusion that the one carcinoma has been transplanted from the other, because already in the normal mucous membrane we find at both places stratified epithelium. One similar carcinoma of the vulva we find recorded by Bang, Stockfleth.

In conclusion we desire to emphasize once more those of our findings that may be of general interest:

1. Through these investigations we shall be enabled to get an insight into the relative frequency of tumors in men and in animals. According to Prussian statistics one death in from thirty to fifty is caused by carcinoma. The average annual death-rate of a standard million human beings (above thirty-five years of age), from 1881 to 1890, in England and Wales, was 1844—*i. e.*, 1.8 per thousand. The annual death-rate per million of all ages, from 1891 to 1895, in England and Wales, was 712—*i. e.*, .7 per thousand. In 1899 we find in cattle one case of carcinoma in fifty thousand. If the statistics, which we intend to continue collecting, should show in the following years a similar proportion, carcinoma in cattle would be about one-fiftieth to one-seventieth less frequent than in man.[1] But certainly these statistics as to the total occurrence of carcinoma in cattle can only be regarded as provisional, if we take into account the great difficulty of making statistics of beginning carcinomatous changes among two and one-half million

[1] A. Newsholme: Vital Statistics, London, 1899.

cattle in one year. However, probably no place offers so good an opportunity for gaining these data as the Chicago Stock Yards. These statistics are of importance, and some way should be found to discover with still greater accuracy the frequency of carcinoma among cattle.

2. The place where carcinoma is found most frequently in cattle is where, through the running of the tears and the motion of the membrana nictitans, foreign bodies entering the conjunctival sac are deposited, and where these foreign bodies can easily be kept back by the hairs of the caruncula. This is favored by the fact that in cattle the caruncula lies somewhat deeper, just between the openings of the superior and inferior lachrymal ducts, and the hairs may also favor the retention of foreign bodies just at this place. But whether the foreign bodies cause the development of a carcinoma by continuous injury, or whether the foreign bodies which are the cause of carcinomata must be certain parasites, our findings do not allow us to decide. So far we have been unable to ascertain from the proprietors whether there exist any peculiar conditions on this Wyoming ranch in regard to the water supply or to the feeding of the animals. We must, therefore, leave it undecided for the present if these observations must be explained by infection from animal to animal or by infection from an external agency. But certain observations previously reported, especially by Behla, in *Centralblatt für Bacteriologie*, 1898, proving in human beings the endemic occurrence of carcinoma in Kalau, seem to be more easily explained by parasitic infection. Behla believes certain organisms of myxamebic character, which infect certain vegetables, to be the cause of this endemic occurrence of carcinoma. But Behla, as others before him, emphasizes the possibility that different carcinomata may be caused by different organisms. Fiessingen, Noel, Armaudet, Sorel, Viguès, and Gueillot,[1] had already, before Behla, reported cases of endemic occurrence of carcinoma in France. Fiessingen and Noel made certain fungi responsible, which are found especially on trees, and which cause there certain tumor-like formations. The proprietors of the ranch with which we have been in communication denied knowledge of any peculiar conditions existing on their farm. Our attention is especially called to these questions by our observation

[1] Cited from Lubarsch u. Ostertag, Ergebnisse der Allgem. Pathologie, 1896.

of the almost endemic occurrence of carcinoma on this ranch, but only by further researches can it be decided if a parasitic cause is present. So far we certainly could not find in the carcinoma any coccidia or yeast-like bodies, the appearance of which could just as well be explained as products of the epithelial cells. Certainly the investigations of the last ten years have at least proved that by simple microscopic examination and staining, the question whether we have to deal with parasites, or with metamorphosed cells or parts of cells, cannot be decided. An interesting discussion of the facts suggesting the parasitic origin of cancer we find in Roswell Park's recent papers—" Cancer as a Parasitic Disease," and " A Further Study into the Frequency and Nature of Cancer."—(*Medical News*, April 1, 1899).

3. We found in our observations a very instructive demonstration that, besides the external exciting causes, there are some other factors of importance in the development of carcinoma, as, for example, the age and possibly also the sex of the animal. In this connection it is well to remember that the statistics of Finkelnburg and Spencer Wells show that in the human race also the female sex is oftener affected by carcinoma than the male, though in the human race carcinoma of the face and extremities seems to be more frequent in males, probably because they are more exposed to injuries than females. But difference in exposure to injury does not apply to cattle; here we find it more frequently in the female. In young animals all these external irritations or parasites, if parasites be present, do not seem to be able to produce carcinoma.

4. Another observation of general interest is the constant absence of metastases in the deeper lymph glands or other organs, although the metastases in the retromaxillary lymph gland very often attain a great size.

The question arises, Does secondary destruction of carcinomatous epithelium in the deeper lymphatic glands take place? Frequently we find in the lymphatic gland necrotic parts and hemorrhages, which prove pathological processes.

Our attention will also be directed in the future to the interesting occurrence of multiple carcinoma. We intend to continue our investigations in these and other directions.

